# Change is never easy: Exploring the transition from undergraduate to dental student in a U.S.-based program

**DOI:** 10.1371/journal.pone.0321494

**Published:** 2025-04-15

**Authors:** Taiana C. Leite, Christine R. Wankiiri-Hale, Nilesh H. Shah, Camille S. Vasquez, Emily M. Pavlowski, Sarah E. Koury, Jia Kim, Kristina M. Ceravolo, Seth M. Weinberg, Zsuzsa Horvath

**Affiliations:** 1 Department of Oral and Craniofacial Sciences, School of Dental Medicine, University of Pittsburgh, Pittsburgh, Pennsylvania, United States of America; 2 Department of Restorative Dentistry and Comprehensive Care, School of Dental Medicine, University of Pittsburgh, Pittsburgh, Pennsylvania, United States of America; 3 Department of Dental Public Health, School of Dental Medicine, University of Pittsburgh, Pittsburgh, Pennsylvania, United States of America; 4 Predoctoral Program, School of Dental Medicine, University of Pittsburgh, Pittsburgh, Pennsylvania, United States of America; University of Maribor, SLOVENIA

## Abstract

The goal of this study was to gain student-centered insights to better understand the challenges of transitioning from undergraduate to dental education. To this end, questionnaires were designed and distributed to incoming dental students, as well as second-, third-, and fourth-year students in the same year for a cross-sectional assessment in 2015/2016. The same questionnaires were also distributed to those same incoming students when they were in their second, third, and fourth years for a longitudinal assessment (2015–2019). There were both open-ended and Likert scale-type questions about expectations (incoming students) and experiences (years 2–4) in dental school compared to undergraduate education. Accordingly, data analysis involved a combination of qualitative and quantitative statistical approaches. Cross-sectional and longitudinal analyses showed that incoming students expected an increased workload in dental school, but also more attention, support, and access to faculty than they received as undergraduates (i.e., they expected a stronger academic support system). All students also reported experiencing more stress and greater difficulty managing their time than expected when compared to their undergraduate experiences. Thus, our study highlights areas of discrepancy between dental students’ initial expectations and their lived experience. Importantly, dental schools can take measures to address these discrepancies, foster a better learning environment, and improve students’ overall experience to help pave a smooth path for students to become successful and well-prepared oral health care providers.

## Introduction

The transition from an undergraduate to a graduate/professional program – such as the transition to dental school – can be a challenging time for incoming students [1]. Students often have pre-conceived notions regarding their upcoming dental school experience and these may be influenced by several sources, including information from current and former dental students and their own experiences in previous programs. One longitudinal study comparing dental students’ expectations to their lived experiences revealed an overall negative shift, suggesting that students initially expected more from their educational environment than what they experienced [2]. This kind of mismatch between expectations and reality can be frustrating, potentially leading to academic and personal struggles, and even mental health issues [3]. This is particularly true for dental students. Several studies report that stress is very prominent among dental students and its sources are mainly related to academic and clinical aspects of dental training [3–5]. If stress levels remain high, they may significantly impact students’ academic performance, physical health, and psychological well-being [4]. An important long-term consequence of stress is burnout. A cross-sectional study showed an alarming figure of 40% of dental students suffering from burnout at an American dental school and reported that prolonged burnout is not sustainable and can lead to depression, self-isolation, and in severe cases, suicidal ideation [6].

Because dental education is inherently challenging and potentially stressful to students, we believe that if incoming students can set realistic expectations, they may be better prepared for the challenges they will face in their education. That preparation may, in turn, reduce potential negative impacts on their education, mental health, and overall wellbeing. For this reason, it is important to evaluate the degree to which incoming dental students’ expectations about dental school match their reality. Thus, the goal of this study was to compare students’ perceptions of their experiences in undergraduate education to expectations and experiences in dental school. To this end, we conducted observational cross-sectional and longitudinal studies comparing expectations and experiences from students in a US-based dental program. By focusing on student-centered insights, we hope to better understand the specific aspects involved in these challenges, help shape preventative strategies, and inform the kinds of resources needed in dental schools to address these issues.

## Materials and methods

### Study participants

Dental students from the four-year predoctoral program at the University of Pittsburgh School of Dental Medicine (UPSDM) were invited to voluntarily complete surveys for two cohorts – a cross-sectional study and a longitudinal study. In all questionnaires, student identity remained anonymous, and each respondent provided an anonymous unique identifier, consisting of the first two letters of their mother’s maiden name and the two digits corresponding to the day of the student’s birth month.

Table 1 shows a schematic for the study outline and response rates. For the *cross-sectional* study, all students in all four years (D1, D2, D3, and D4) in the 2015–16 academic year who were present at the time of distribution of questionnaires were invited to participate. Enrolled D1 students were invited to answer the questionnaire during orientation early in the fall semester of 2015. In that same school year, D2, D3, and D4 students were invited to participate during class. For the *longitudinal* study, the students from the class of 2019 were surveyed four times, once every year during their education, also during class. Recruitment took place between August 19, 2015, and April 1, 2019.

**Table 1:** Study design and response rates.

	CROSS-SECTIONAL			
	2015/2016	2017	2018	2019
LONGITUDINAL	**D1** Class of 2019 ***Baseline**** questionnaire* Response rate: 100% (n=78)	**D2 **Class of 2019 ***Follow-up**** questionnaire* Response rate: 97.3% (n=74)	**D3 **Class of 2019 ***Follow-up**** questionnaire* Response rate: 84.2% (n=64)	**D4 **Class of 2019 ***Follow-up**** questionnaire* Response rate: 76% (n=69)
	**D2 **Class of 2018 *Questionnaire ****2*** Response rate: 100% (n=76)			
	**D3 **Class of 2017 *Questionnaire ****2*** Response rate: 76.6% (n=59)			
	**D4 **Class of 2016 *Questionnaire ****2*** Response rate: 70% (n=56)			

Students were invited to complete paper questionnaires in person. These questionnaires were distributed to all students present in the classroom at all time points. The purpose of the survey was explained to students and that their participation was completely voluntary. At that point, completion of the survey was taken as informed consent. This study was approved as exempt by the University of Pittsburgh Human Research Protection Office (PRO15070414).

### Survey questionnaires

The questionnaires contained two sections. The *first section* included questions about undergraduate and current (when applicable) academic achievements, such as GPA and undergraduate institution. The *second section* included the bulk of the survey, with eight questions comparing expectations or experiences between undergraduate and dental school. Three of these questions were open-ended and addressed differences between undergraduate and dental school instructors, classes, and experiences outside the classroom. The remaining five were Likert scale-type questions regarding differences between undergraduate and dental school overall preparedness, time management, workload, stress, and academic support. Questions were carefully chosen by a panel of experts (CWH and ZH) based on their experience in the areas of teaching, clinical education, admissions, and student affairs, and on Appleby’s 2014 study [7], which examined the expectations and differences between high school and college. Open-ended questions were deliberately used to allow students to respond with spontaneity.

Three different questionnaires were distributed, depending on the study group (Table 1). The *first questionnaire*, henceforth referred to as *baseline questionnaire*, was distributed to incoming D1 students, who were included in both cross-sectional and longitudinal cohorts, asking questions of both sections. Specifically, its second-section questions asked students to compare their *experiences* as undergraduates to their *expectations* in their upcoming dental school education. The *second questionnaire* was distributed to the cross-sectional cohort, i.e., D2, D3, D4 students, at the same time point as the first questionnaire, and also included questions of both sections. Specifically, its second-section questions asked students to compare their *experiences* as undergraduates to their *experiences* as dental students. The *third questionnaire*, henceforth referred to as *follow-up questionnaire* was utilized only for the longitudinal cohort including only second-section questions comparing their *experiences* as undergraduates to their *experiences* as dental students.

The complete study questionnaires are provided as the supplemental materials (S1–S3 File).

### Data analysis

Written responses were transformed to electronic versions using Qualtrics™ and exported into Excel spreadsheets for subsequent analyses. For open-ended questions in both cross-sectional and longitudinal cohorts, a coding system was created to group common themes (the five most common themes are listed and explained in Table 2). Two calibrated, independent coders (CV, EP) analyzed each response separately and later reconvened to create a final combined dataset. The categorized open-ended question responses were displayed in percentages and summarized with descriptive statistics. Less frequently mentioned themes included frequency of exams, responsibility and professionalism, and social environment of learning (classes); less access to faculty and professionalism (instructors); developing interpersonal skills, activities related to dentistry, and independence (non-classroom experiences). These scarcely mentioned themes were not included in our subsequent analyses.

**Table 2:** List of categories for students’ responses to open-ended questions regarding expected/experienced differences between dental and undergraduate educations for both cross-sectional and longitudinal studies.

DENTAL SCHOOL COMPARED TO UNDERGRADUATE CLASSES	DENTAL SCHOOL COMPARED TO UNDERGRADUATE INSTRUCTORS	DENTAL SCHOOL COMPARED TO UNDERGRADUATE NON-CLASSROOM EXPERIENCES
**Increased workload:** increase of content and time during school hours.	**More personalized attention/more support:** increased one-on-one attention, faculty are more invested in helping students reach their goals/become successful; overall closer relationship between students and faculty.	**Professionalism/more dental-related experiences:** use of maturity, networking, emotional investment, and good judgement needed to succeed in the dental profession/ increase in dental-related activities.
**More depth/more difficult:** content is expected to be learned in more depth and to be more difficult; dental school is overall harder/more intense.	**More access to faculty:** increased availability of faculty to meet with students in person (office hours, class, lab, etc.) or online (email).	**Time management/less free time:** higher need to use time more productively, less time to complete non-dental related activities (not in a social setting/less sleep).
**Faster pace:** increased rate of learning the necessary material.	**Faculty demand more from students:** faculty have higher expectations for students’ work and success.	**Closer with classmates:** closer friendships among students, more time spent with classmates.
**Relevance to dentistry:** content taught and learned is more related to dentistry.	**Faculty are more knowledgeable/ experienced:** faculty are well informed/have more experienced backgrounds in the dental field.	**Less social time:** less time participating in non-dental school related activities in a social setting with friends/people.
**Time management/ more time consuming:** more instances to use time more productively/school takes up more time.	**Faster paced teaching/different teaching style:** faculty teach classes at a faster rate/teach classes in a different style.	**Increased stress level/ expectations:** more stressful environment/more demanding lifestyle.

As for Likert score-like questions, students of both cross-sectional and longitudinal cohorts responded to expected/experienced differences between dental school and undergraduate pertaining to preparedness for dental school, workload, ability to manage time, stress level, and rate of academic support, using Likert scale-like scores. For the cross-sectional study, these data were analyzed using Stata™ (v.17) software. The Shapiro-Wilk test for normality showed that many responses did not follow normal distribution. We, therefore, utilized the Kruskal-Wallis test to analyze cross-sectional cohort responses, followed by the Wilcoxon Rank-Sum test for paired comparisons, when a significant difference was found. To test for potential confounders for the Likert scale-type responses, we ran a multiple linear regression model, using students’ responses for the questionnaires’ first sections, regarding academic performance. These covariates included undergraduate/graduate GPA, having held a job as an undergraduate/graduate, undergraduate/graduate research experience, age upon entrance at dental school, and Barron’s college ranking [8] based on name of college attended for undergraduate education. Mixed-effects regression modeling was utilized to analyze the longitudinal cohort. We modeled each Likert scale-type response longitudinally and examined the impact of year while adjusting for potential confounding variables related to academic performance – undergraduate/graduate GPA, having held a job as an undergraduate/graduate, and undergraduate/graduate research experience (age and college rankings were not included, as they did not change, because the same students were analyzed).

A complete case analysis was performed for these longitudinal data, excluding responses from students who were absent from one or more questionnaires, and those with duplicate anonymous unique identifiers. Because the questionnaires were anonymous, it was not possible to track down the cause of absence for each individual case; possible causes include: students chose not to complete the questionnaire, were absent from school that day, or did not use consistent unique identifiers throughout the years. Whichever the case, we are comfortable assuming that these data were missing completely at random. Significance was considered for p-values lower than 0.05 for Kruskal-Wallis tests and regression models, and 0.008 for Wilcoxon Rank-Sum tests, using Bonferroni correction for multiple paired comparisons (0.05 divided by 6 comparisons, resulting in 0.008).

## Results

### Response rates

The response rate varied depending on the different groups, because each questionnaire was administered to different classes (Table 1). The response rate was calculated by the dividing number of questionnaires filled-out by the number of students in the class roster. After accounting for missing data, there were 46 participants with non-duplicated responses available across the four time points for the longitudinal dataset. The raw questionnaire datasets used in our analysis are provided as supplemental materials (S4–S12 File).

### Cross-sectional study outcomes

#### Open-ended questions.

**Fig 1:**
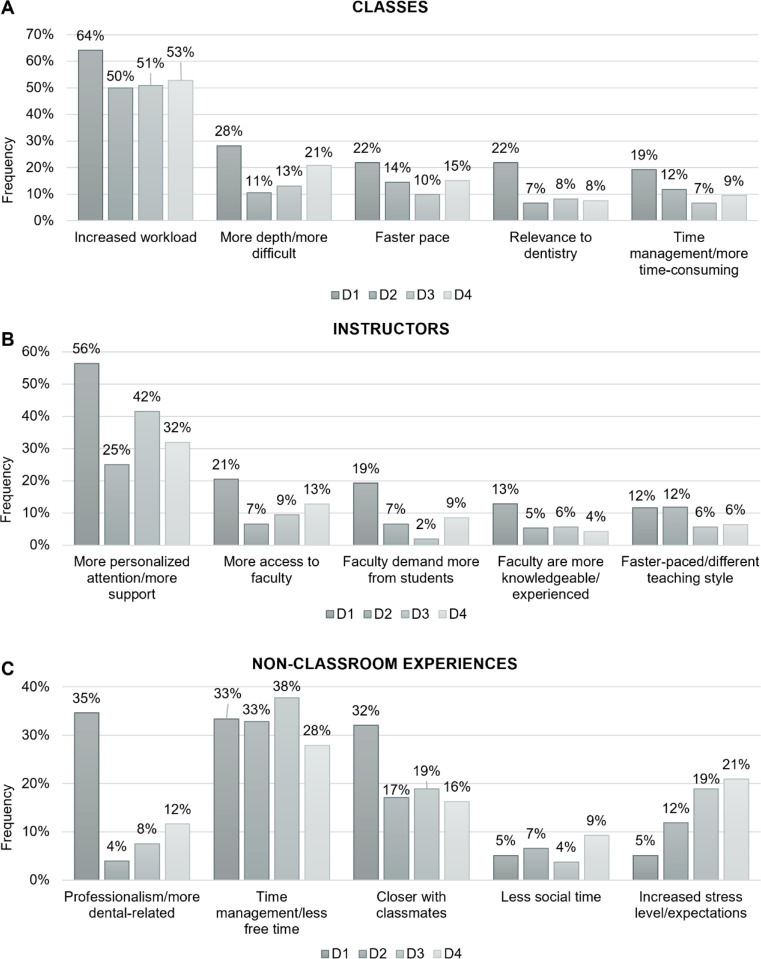
*Cross-sectional* study open-ended question responses. D1 (class of 2019), D2 (class of 2018), D3 (class of 2017), and D4 (class of 2016) students’ responses to expected/experienced differences between **(A)** undergraduate classes and dental school classes, **(B)** undergraduate instructors and dental school instructors, and **(C)** undergraduate non-classroom experiences and non-classroom experiences in dental school surveyed in 2015.

Open-ended question responses were grouped into categories and the five most common categories mentioned by the incoming D1 class (Table 2) were selected for comparisons among different classes in our descriptive analyses (Fig 1).

Regarding responses to differences between *classes* in dental school versus undergraduate, most D1 students (64%) expected there to be increased workload in dental school (Fig 1A). Proportions remained high but dropped more than 10% in D2 through D4 reflecting their lived experience with increased workload in dental school. For the other four categories, a common pattern emerged with a greater number of D1 students expecting increased depth and difficulty, a faster pace, more relevance to dentistry, and a more difficultly with time management compared to the experiences of their D2, D3, and D4 colleagues.

The gap between D1 students’ expectations and more senior students’ lived experience also extended to *instructors* (Fig 1B). The most jarring discrepancy related to expectations of more personalized attention and more support, with a 31% difference between D1 expectations and D2 experience.

Response relating to *non-classroom-related experiences* were more complex (Fig 1C). While expectations around professionalism and having close relationships with classmates showed the same pattern of gross discrepancy with lived experience as those reported above, the gap narrowed considerably for time management and time for social life. Notably, responses concerning increased stress levels and expectations was initially low in D1 students but continued to rise each year through D4.

#### Likert scale-type questions.

The linear regression model did not reveal any potential confounders impacting students’ responses when accounting for academic performance.

**Fig 2:**
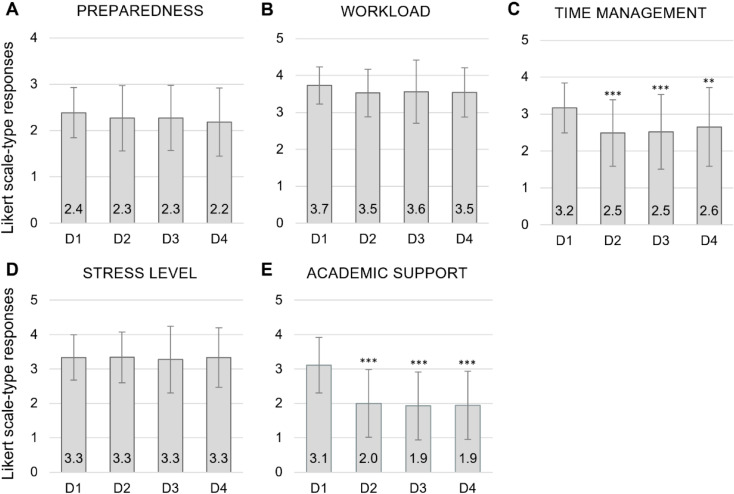
*Cross-sectional* study Likert scale-type responses. D1 (class of 2019), D2 (class of 2018), D3 (class of 2017), and D4 (class of 2016) students’ responses to expected/experienced differences between dental school and undergraduate regarding **(A)** preparedness for dental school, **(B)** workload, **(C)** ability to manage time, **(D)** stress level, and **(E)** rate of academic support. For *preparedness*, 0 = Very inadequate, 1 = Somewhat inadequate, 2 = Somewhat adequate, and 3 = Very adequate. For *workload*, 0 = Much less, 1 = Less, 2 = About the same, 3 = More, and 4 = Much more. For *time management*, 0 = Much worse, 1 = Worse, 2 = About the same, 3 = Better, and 4 = Much better. For *stress level*, 0 = Much lower, 1 = Lower, 2 = About the same, 3 = Higher, and 4 = Much higher. For *academic support system*, 0 = Much worse, 1 = Worse, 2 = About the same, 3 = Better, and 4 = Much better. Data displayed as mean and standard deviation. **P-value < 0.008 and ***p-value < 0.0001 of D2, D3, and D4 in comparison to D1, as determined by Wilcoxon Rank-Sum test.

Of the five Likert scale-type questions, Kruskal-Wallis tests showed a statistically significant difference for *time-management* (p < 0.0001) and *academic support* (p < 0.0001), but no significant differences between responses for overall *preparedness* for dental school, the *workload* they will face, and the level of *stress* they will endure. Upon further investigation, Wilcoxon Rank-Sum tests showed these differences were present between D1 students and all other classes (D2, D3, and D4) (Fig 2), with D1 students expecting to have a significantly greater ability to manage time (Fig 2C) and, notably, expecting to have a higher level of academic support (Fig 2E).

### Longitudinal study outcomes

#### Open-ended questions.

**Fig 3:**
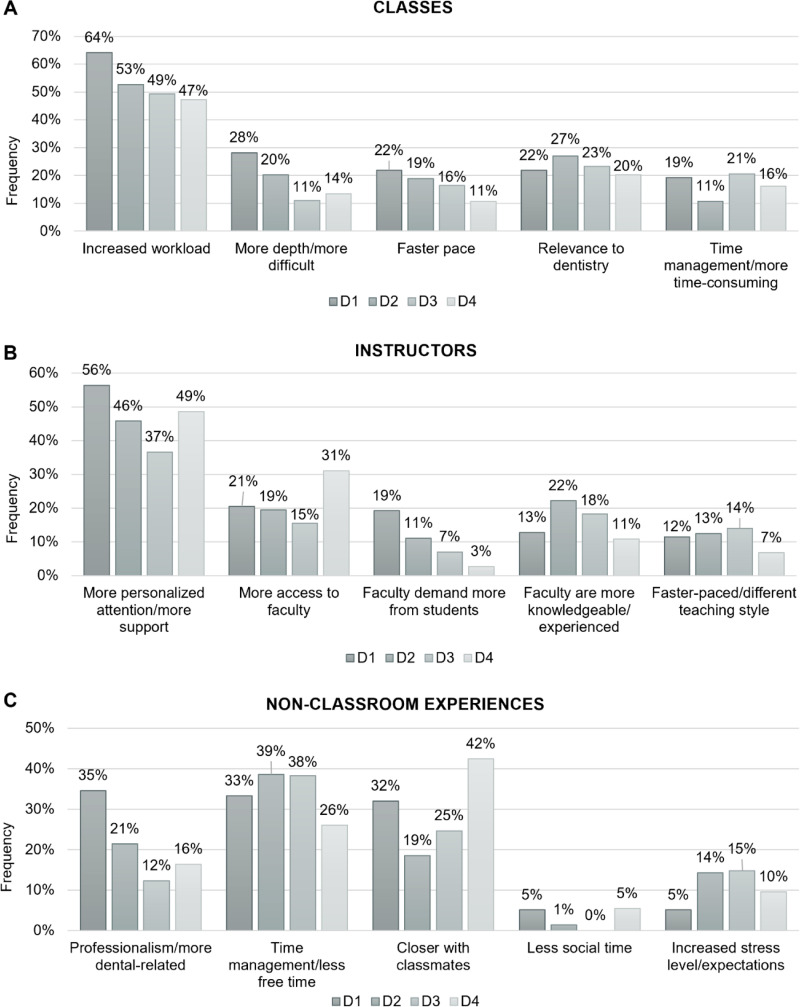
*Longitudinal* study open-ended question responses. Class of 2019 (D1, D2, D3, and D4) students’ responses to expected/experienced differences between **(A)** undergraduate classes and dental school classes, **(B)** undergraduate instructors and dental school instructors, and **(C)** undergraduate non-classroom experiences and non-classroom experiences in dental school throughout all four years of dental school.

Again, the five most common categories mentioned by the D1 class were selected for comparison among different classes in our descriptive analyses (Fig 3).

Regarding the class of 2019’s expectations about *classes* in dental school compared to what they experienced as undergraduates, responses expressing concern for increased workload dropped as their education progressed (from 64% to 47%) (Fig 3A). A similar pattern of reduction was observed for concerns about increased depth/difficulty and faster pace. In contrast, D1 expectations and D2-D4 experiences concerning relevance to dentistry and time management concerns were more similar.

As for questions about *instructors*, the pattern of responses was variable across the four years. While fewer students mentioned receiving more personalized attention and support in their D2-D4 years compared to their initial D1 year expectations, the discrepancy was less evident than in the cross-sectional study (Fig 3B). D1 expectations regarding more access to faculty fluctuated from having similar percentages at the D2 and D3 stage, to having a higher percentage of responses by D4. Responses indicating that faculty would be more knowledgeable and experienced were higher at D2 than initially expected at D1, but then fell gradually during the D3 and D4 years. Lastly, responses referencing teaching styles being different and faster-paced had somewhat similar proportions across the four years (around 12%).

Expectations about *non-classroom-related experiences* in dental school compared to undergraduate were mostly similar to those observed for the cross-sectional study, with a few exceptions. Responses regarding having more professionalism and dental-related experiences showed a drop between D1 and later years (Fig 3C), similar but less drastic than we observed for the cross-sectional study. Notably, we observed the same drop between D1 and D2/3 concerning being closer with classmates, but this pattern was then reversed in D4.

#### Likert scale-type questions.

**Fig 4:**
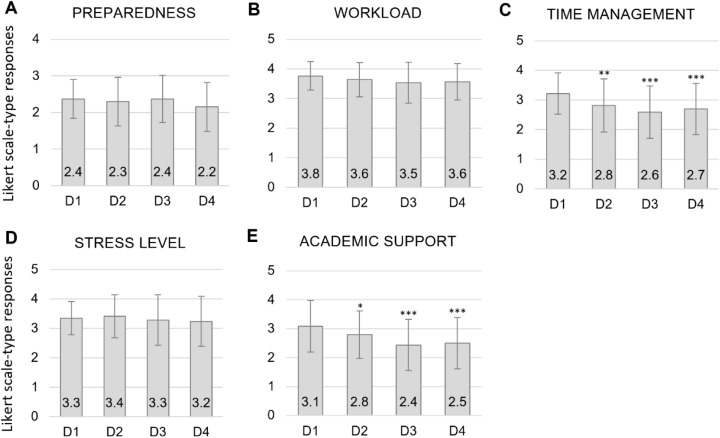
*Longitudinal* study Likert scale-type responses. D1, D2, D3, and D4 (class of 2019) students’ responses to expected/experienced differences between dental school and undergraduate regarding **(A)** preparedness for dental school, **(B)** workload, **(C)** ability to manage time, **(D)** stress level, and **(E)** rate of academic support. For *preparedness*, 0 = Very inadequate, 1 = Somewhat inadequate, 2 = Somewhat adequate, and 3 = Very adequate. For *workload*, 0 = Much less, 1 = Less, 2 = About the same, 3 = More, and 4 = Much more. For *time management*, 0 = Much worse, 1 = Worse, 2 = About the same, 3 = Better, and 4 = Much better. For stress level, 0 = Much lower, 1 = Lower, 2 = About the same, 3 = Higher, and 4 = Much higher. For *academic support system*, 0 = Much worse, 1 = Worse, 2 = About the same, 3 = Better, and 4 = Much better. Data displayed as mean and standard deviation. *p-value < 0.05, ** p-value < 0.008 and ***p-value < 0.0001 of D2, D3, and D4 in comparison to D1, as determined by the mixed-effects regression modeling.

Similar to what was observed in the cross-sectional Likert scale-type results, in the longitudinal cohort there were also no significant differences between responses for overall *preparedness*, *workload*, and *stress levels*. Also mirroring the cross-sectional results, there were significant differences between D1 expectations and what they report in years 2, 3, and 4 for *time management* (p < 0.0001) and level of *academic support* (p < 0.0001) (Fig 4 and Table 3). Responses indicated expectation of less difficulty in managing time compared to their time as undergraduates (Fig 4C). The same went for expectations regarding academic support versus what they received throughout their dental education (Fig 4E). Interestingly, when analyzing for potential confounders regarding academic performance, the statistical significance was not impacted for academic support, but disappeared for time management (Table 4).

**Table 3:** Unadjusted coefficients (95% CI) from mixed-effects linear regression for Likert score outcome. Coefficients represent the change in score compared to Year 1.

	PREPAREDNESS	WORKLOAD	TIME MANAGEMENT	STRESS LEVEL	ACADEMIC SUPPORT
YEAR 1	Reference	Reference	Reference	Reference	Reference
YEAR 2	−0.07 (−.27, 0.13)	−0.12 (−0.30, 0.07)	−0.41 (−0.69, −0.13)*	0.07 (−0.18, 0.33)	−0.29 (−0.57, −0.01)*
YEAR 3	0.00 (−0.20, 0.20)	−0.23 (−0.42, −0.05)*	−0.63 (−0.91, −0.35)*	−0.07 (−0.32, 0.18)	−0.65 (−0.93, −0.38)*
YEAR 4	−0.22 (−0.42, −0.02)*	−0.20 (−0.38, −0.01)*	−0.52 (−0.80. −0.24)*	−0.11 (−0.36, 0.14)	−0.59 (−0.86, −0.31*)

* p < 0.05.

**Table 4:** Coefficients (95% CI) from mixed-effects linear regression for Likert score outcome, adjusted for potential baseline confounding variables. Coefficients represent the change in score compared to Year 1.

	PREPAREDNESS	WORKLOAD	TIME MANAGEMENT	STRESS LEVEL	ACADEMIC SUPPORT
YEAR 1	Reference	Reference	Reference	Reference	Reference
YEAR 2	−0.10 (−0.43, 0.22)	−0.06 (−0.25, 0.13)	−0.12 (−0.54, 0.30)	0.15 (−0.26, 0.56)	−0.14 (−0.601, 0.32)
YEAR 3	−0.07 (−0.37, 0.23)	−0.06 (−0.28, 0.16)	−0.41 (−0.81, −0.02)*	0.05 (−0.33, 0.43)	−0.59 (−1.02, −0.16)*
YEAR 4	−0.29 (−0.59, 0.01)	−0.30 (−0.56, −0.05)*	−0.28 (−0.67, 0.12)	−0.03 (−0.41, 0.35)	−0.47 (−0.90, −0.04)*
WORK EXPERIENCE	0.10 (−0.12, 0.33)	−0.06 (−0.25, 0.13)	0.13 (−0.17, 0.43)	0.12 (−0.17, 0.40)	0.14 (−0.19, 0.46)
RESEARCH EXPERIENCE	−0.22 (−0.47, 0.03)	−0.06 (−0.28, 0.16)	0.15 (−0.18, 0.49)	0.04 (−0.28, 0.36)	0.01 (−0.36, 0.37)
GPA	0.09 (−0.03, 0.22)	−0.11 (−0.21, 0.00)*	0.10 (−0.06, 0.26)	−0.09 (−0.25, 0.06)	0.01 (0.17, 0.19)

* p < 0.05.

## Discussion

The main purpose of this study was to investigate pre-doctoral students’ expectations and experiences throughout the dental program in comparison to their undergraduate experiences. The cross-sectional cohort was intended to give a snapshot of expectations and experiences across the classes for a given year, whereas the longitudinal cohort was intended to allow observation of the same group of students over time. Both cohorts had similar differences between what incoming students expected and what was experienced in later years during their education. Of note, D1 students based their expectations solely on what they had heard about dental school, as they hadn’t had any dental curricular experience at the time when questionnaires were distributed.

Open-ended question results for both cohorts showed that D1 students expected increased workload, paralleling their more senior peers’ reports. Another study also found that dental students reported a heavier workload when compared to their undergraduate experience [9]. However, in our study, other D1 expectations regarding classes were reported in lower proportions by D2, D3, and D4 students, including more depth and more difficulty, faster pace, relevance to dentistry, and more difficulty in managing time. The decrease in these proportions is possibly due to students’ ability to adjust during their education and could also be attributed to the fact that clinic time increases throughout the curriculum and students have significantly fewer (or no) didactic classes by the time they reach their fourth year. As a result, they report encountering fewer class-related issues in these final years. The decrease may also be related to the culture of dental education being known as inherently challenging [4]. In other words, students may have an expectation that their education will be difficult, but once they are enrolled, they face fewer challenges than initially expected.

The cross-sectional open-ended responses also showed that incoming students expected more attention, support, and access to faculty than they received as undergraduates, whereas their senior peers mentioned these themes in smaller proportions. The longitudinal data showed similar overall trends, though the disparity was less evident. Previous studies indicate comparable trends, with dental students perceiving that faculty were less helpful and supportive than anticipated, with antiquated teaching methodologies and rigid hierarchical relationships offered as a possible explanation [10,11]. Even today, traditional teaching methods are still widely prevalent in dental education; however, students’ prior educational experience may have changed to include more up-to-date instructional strategies, priming them for potential disappointment when entering dental education. Additionally, students’ rationale for expecting more attention may be due to the apprenticeship model characteristic of health professional education. Moreover, depending on their undergraduate experience (possibly at a larger institution), students may expect increased faculty attention due to a more favorable student-faculty ratio. Conversely, from the faculty’s perspective, dental students may be viewed as adults, and more mature than undergraduate students, therefore not requiring as much attention.

Another noteworthy finding from our qualitative data is that D1 students’ expectations regarding having more professionalism and dental-related experiences outside the classroom had a higher percentage of responses compared to their senior peers’. A possible explanation for this discrepancy could be that D2, D3, and D4 students have an implicit understanding that opportunities for profession-related exposures as dental students will take place outside of the curriculum, and therefore, do not consider including this aspect in their responses when asked about their non-classroom-related experiences.

Students’ responses mentioning being closer to classmates showed different patterns in the cross-sectional and longitudinal studies. In our cross-sectional data, student’s initial expectations were higher compared to the reported experiences of D2-D4 classes. This could be due to intrinsic difference among the different classes, and we do not know how the experience of any single class might have changed over time. We can glean some additional insights, however, from our longitudinal data. Here we saw a similar pattern in higher expectations for the incoming D1 class compared to their experiences in their D2 and D3 years, but this changed dramatically in their D4 year, rising even higher than their initial expectations at the start of dental school. A possible explanation could be that the initial camaraderie expected in D1 was negatively impacted due to stressors and the high degree of competitiveness characterizing their D2 and D3 years. In contrast, the D4 year represents a new stage of their dental education marked by less competition as most students have fulfilled the majority of their requirements and placed into a residency program or secured their first job. Another recent study from the University of Hong Kong reported overall high rates of dental students having “a positive relationship with their classmates” [12], but it is difficult to compare our results due to cultural and curricular differences and the fact that their study did not present these data broken down by class year or assess attitudes longitudinally.

For open-ended questions, most students also reported more stress than anticipated, although the high stress level aligns with the expectation reported in the Likert scale-type questions. Though far from ideal, this is expected and has been previously reported as an inherent component of dental or health profession educations [3,4,13,14].

As for Likert scale-type questions, both cohorts reported having more difficulty managing time than expected when compared to their undergraduate experiences. However, for the longitudinal group, the effect was attenuated when factoring in academic performance confounders. Considering that the longitudinal group is more uniform, it could be interpreted that more adept students would be able to manage their time more efficiently and would therefore have more realistic expectations. On the other hand, it could also be that this result was impacted by sample size, which was limited to students who were present in all four time points, and model complexity, rather than the impact of confounders. Regardless, a good management of time is desirable not only because the workload requires it, but also because it has been shown to help with coping with stress [14] and maintaining adequate work-life balance [13].

Importantly, recurring responses in both cohorts indicated that students expected to have a stronger academic support system than they did as undergraduates, but that D2-D4 experiences fall below these expectations. The lack of academic support was also pointed out by dental students in other studies, including a recent cross-sectional investigation [10]. It should, however, be pointed out that this study was conducted prior to the COVID-19 pandemic. During and after the pandemic, many institutions, including the UPSDM, implemented a number of measures to improve students’ experience, with special focus on support and mental health [15,16].

Several such measures can be taken to address the results discussed above, foster a better learning environment, and improve students’ overall experience in a dental school [12,14]. Dental schools may use the data collected to better curate their curriculum, extra-curricular offerings, and faculty training (which would address some of the recurring responses by students) to create a more supportive environment [11,13]. To prevent frustration, stress (which was also mentioned in students’ responses), and burnout, dental schools may consider offering early detection/prevention programs and interventions to foster psychological safety amongst students [17–19]. In this regard, one study found that medical and dental students thought early psychological assessment and counseling to be very important for students [20]. With the popularity of social media, virtual group therapy may provide students with an alternative source of help [6]. Examples of such measures have already been implemented or enhanced at the UPSDM after this study was conducted, and include peer tutoring programs, hiring of a full-time Licensed Professional Counselor, identifying an ombudsperson, a meditation room, earlier extended onboarding, student focus groups, among many others. Finally, to avoid overwhelming students, it is key to properly curate all resources and communicate to students their availability, and to clarify how they can properly be used to their best advantage [12].

As a future direction, it may be worthwhile to redistribute questionnaires to students to measure effects of implemented actions, gauge future changes, and assess whether the discrepancy between expectations and experiences will be significantly reduced. Similar to other health professions, transitions at other time points in dental education, e.g., from predoctoral to residency education, may also be worth investigating [21].

## Limitations

Our study only included responses from a single institution, which poses a limitation, because it might have introduced possible biases pertaining to geographical location, philosophical aspects of the university, and cultural background of students. However, it should be noted that our students have a similar profile when compared to the national data of the same period, so it is reasonable to assume that our findings are generalizable [22]. Nonetheless, single classes may have individual characteristics that set them apart from other classes, which might also have introduced bias to the cross-sectional study. We were also limited by sample size, as there is a restricted number of students enrolled in each year, as well as uncontrollable issues, such as absences and duplicate unique identifiers. Repeating similar studies in other institutions could potentially provide more reliable conclusions and more broadly applicable recommendations. Another limitation was the fact that our questionnaires were not validated prior to distribution. However, we considered them robust, as they were based on the literature [7] and were revised by a panel of experts. Furthermore, while open-ended questions were intentionally included because they offer rich data and allow the participants to include any relevant experience, they limit conclusions, because students were not specifically prompted to report their experiences in specific themes, such as those mentioned in D1 responses.

## Conclusions

This study provides student-centered insights to better understand specific challenges when transitioning from undergraduate to dental education by documenting dental students’ expectations and experiences in a professional health education environment. We found that some of the students’ initial expectations at the start of their first year differed from the experiences of students in later years. Some of these discrepancies were in key areas, related to managing workloads or the degree of academic support available. Dental schools should consider implementing measures to address these issues and help pave a smoother path from admission to graduation.

## Supporting information

S1 File Questionnaire 1-baseline-incomming Survey. (PDF)

S2 File Questionnaire 2-cross-sectional Survey. (PDF)

S3 File Questionnaire 3-Follow-up-longitudinal Survey. (PDF)

S4 File Likert cross-sectional data. (XLSX)

S5 File Likert longitudinal data. (XLSX)

S6 File Open ended-cross-sectional and longituinal-D1. (XLSX)

S7 File Open ended-cross-sectional-D2. (XLSX)

S8 File Open ended-cross-sectional-D3. (XLSX)

S9 File Open ended-cross-sectional-D4. (XLSX)

S10 File Open ended-longituinal-D2. (XLSX)

S11 File Open ended-longituinal-D3. (S11)

S12 File Open ended-longituinal-D4. (XLSX)
