# Supplementary material for: Change is never easy: Exploring the transition from undergraduate to dental student in a U.S.-based program
Source: PLoS One. 2025 Apr 15;20(4):e0321494. doi: 10.1371/journal.pone.0321494 (PMC11999116; doi:10.1371/journal.pone.0321494)
Supplement: S1 File — (PDF) [file pone.0321494.s001.pdf]

Appendix A: Survey - Dental Students' Perception of Undergraduate and Pre-doctoral Experience Incoming Students

The goal of this study is to help students at dental schools have more realistic expectations and be better prepared for the academic expectations and experience during their education in the dental pre-doctoral program. Based on the results from this study, faculty and administrators at dental schools will be able to identify areas in which schools can provide support to the struggling students to adapt to the expectations in the dental school environment. Responses will be tracked longitudinally via the anonymous unique identifier. This study was approved by the University of Pittsburgh IRB (IRB #: PRO15070414) on 8/13/2015.

Anonymous Unique Identifier
First two letter of you mother's maiden (sur)name: ___ ___
Day of the month you were born (add leading zero if it's one digit): ___ ___
Example: Mary Smith (maiden name: Mary Kline): **K L**
March 4, 1995: **0 4**

---

1. **What was your overall undergraduate GPA?** ❑ 2.75 - 3.0 ❑ 3.25 - 3.5 ❑ 3.0 - 3.25 ❑ 3.5 - 4.0

2. **Did you attend Community College at any point in your education? If yes, how many semesters/terms?** ❑ Yes ❑ No

3. **Which college did you attend for your undergraduate degree? (please provide the official name, state and country)**

____

4. **What was your major(s)?** ❑ n/a

5. **What was your minor(s)?** ❑ n/a

6. **What degree(s) do you hold (mark all that apply)? If other, please specify.**
❑ BS ❑ MA ❑MsED ❑ MD
❑ BA ❑ MBA ❑ PhD ❑BDS/MDS
❑ MS ❑ MPH ❑ JD ❑other

7. **What are the most important differences you expect to experience between your undergraduate classes and dental school classes?**

____
____
____

8. **What are the most important differences you expect to experience between your undergraduate instructors and your dental school instructors?**

____
____
____

9. **What are the most important differences you expect to experience between your undergraduate experiences and your dental school experiences that are not related to classes?**

____
____
____

10. **What was the average (academic average) on your DAT score?** ❑ 16 -17 ❑ 21 – 23 ❑ 27 – 30 ❑ 18 - 20 ❑ 24- 26 ❑ n/a

11. **What undergraduate education/extracurricular experiences did you take? (please specify)** ❑ n/a
(e.g. DAT prep courses, summer dental enrichment courses, pre-dental club, health related mission trips, etc.)

____
____

Please turn over ➔

12. **Do you have a dental professional in the family, if so what is their profession?** ❑ Yes ❑ No

❑Dentist, ❑Dental Hygienist, ❑Dental Assistant, ❑Dental Technician, ❑Other (please specify)

____

13. **At what age did you start Pitt Dental school?**
   - ❑ < 21
   - ❑ 21-23
   - ❑ 24-26
   - ❑ 27-30
   - ❑ >30

14. **How would you rate your preparedness for dental school?**
   - ❑ Very adequate
   - ❑ Somewhat adequate
   - ❑ Somewhat inadequate
   - ❑ Very inadequate

15. **How do you expect the work load to be in dental school compared to undergraduate?**
   - ❑ Much More
   - ❑ More
   - ❑ About the same
   - ❑ Less
   - ❑ Much less

16. **How do you expect your time management ability to be in dental school compared to undergraduate?**
   - ❑ Much better
   - ❑ Better
   - ❑ About the same
   - ❑ Worse
   - ❑ Much worse

17. **How do you expect your stress level to be in dental school compared to undergraduate?**
   - ❑ Much higher
   - ❑ Higher
   - ❑ About the same
   - ❑ Lower
   - ❑ Much Lower

18. **How do you expect the academic support system to be in dental school compared to undergraduate?**
   - ❑ Much better
   - ❑ Better
   - ❑ About the same
   - ❑ Worse
   - ❑ Much worse

19. **Did you have work experience prior to dental school?** ❑ Yes ❑ No

   If **yes**, was it in a dental setting? (please specify type)

   ❑ Yes, in dental; experience: ____

   ❑ No, not in dental; experience: ____

20. **Did you have research experience prior to dental school?** ❑ Yes ❑ No

   If **yes**, was it in a dental setting? (please specify type)

   ❑ Yes, in dental; experience: ____

   ❑ No, not in dental; experience: ____
