# Supplementary material for: Change is never easy: Exploring the transition from undergraduate to dental student in a U.S.-based program
Source: PLoS One. 2025 Apr 15;20(4):e0321494. doi: 10.1371/journal.pone.0321494 (PMC11999116; doi:10.1371/journal.pone.0321494)
Supplement: S2 File — (PDF) [file pone.0321494.s002.pdf]

Appendix B: Survey - Dental Students' Perception of Undergraduate and Pre-doctoral Experience
Dental Student (second, third and fourth year dental students in the first year of the study)

The goal of this study is to help students at dental schools have more realistic expectations and be better prepared for the academic expectations and experience during their education in the dental pre-doctoral program. Based on the results from this study, faculty and administrators at dental schools will be able to identify areas in which schools can provide support to the struggling students to adapt to the expectations in the dental school environment. This study was approved by the University of Pittsburgh IRB (IRB #: PRO15070414) on 8/13/2015.

Please indicate in which class you are ❑ **Class of 2016** ❑ **Class of 2017**

1. **Which college did you attend for your undergraduate degree? (please provide the official name, state and country)**

____

2. **Did you attend Community College at any point in your education? If yes, how many semesters/terms?** ❑ Yes ❑ No

3. **What was your major(s)?** ❑ n/a

4. **What was your minor(s)?** ❑ n/a

5. **What degree(s) do you hold (mark all that apply)? If other, please specify.**
❑ BS ❑ MA ❑MsED ❑ MD
❑ BA ❑ MBA ❑ PhD ❑BDS/MDS
❑ MS ❑ MPH ❑ JD ❑other

6. **What are the most important differences you have experienced between your undergraduate classes and dental school classes?**

____
____
____

7. **What are the most important differences you have experienced between your undergraduate instructors and your dental school instructors?**

____
____
____

8. **What are the most important differences you have had between your undergraduate experiences and your dental school experiences that are not related to classes?**

____
____
____

9. **What was your overall undergraduate GPA?**
❑ 2.75 - 3.0 ❑ 3.25 - 3.5
❑ 3.0 - 3.25 ❑ 3.5 - 4.0

10. **What was the average (academic average) on your DAT score?**
❑ 16 -17 ❑ 21 – 23 ❑ 27 – 30
❑ 18 - 20 ❑ 24- 26 ❑ n/a

11. **What undergraduate education/extracurricular experiences did you take? (please specify)** ❑ n/a
(e.g. DAT prep courses, summer dental enrichment courses, pre-dental club, health related mission trips, etc.)

____
____

12. **Do you have a dental professional in the family, if so what is their profession?** ❑ Yes ❑ No
❑Dentist, ❑Dental Hygienist, ❑Dental Assistant, ❑Dental Technician, ❑Other (please specify)

____

Please turn over ➔

13. **At what age did you start Pitt Dental school?** ❑ < 21 ; ❑ 21-23; ❑ 24-26 ; ❑ 27-30 ; ❑ >30

14. **How would you rate your preparedness for dental school?**
- ❑ Very adequate
- ❑ Somewhat adequate
- ❑ Somewhat inadequate
- ❑ Very inadequate

15. **How would you rate the work load in dental school compared to undergraduate?**
- ❑ Much More
- ❑ More
- ❑ About the same
- ❑ Less
- ❑ Much less

16. **How would you rate your ability to manage your time in dental school compared to undergraduate?**
- ❑ Much better
- ❑ Better
- ❑ About the same
- ❑ Worse
- ❑ Much worse

17. **How would you rate your stress level in dental school compared to undergraduate?**
- ❑ Much higher
- ❑ Higher
- ❑ About the same
- ❑ Lower
- ❑ Much Lower

18. **How would you rate the academic support system in dental school compared to undergraduate?**
- ❑ Much better
- ❑ Better
- ❑ About the same
- ❑ Worse
- ❑ Much worse

19. **Did you have work experience prior to dental school?** ❑ Yes ❑ No

If **yes**, was it in a dental setting? (please specify type)
❑ Yes, in dental; experience: ____

❑ No, not in dental; experience: ____

20. **Did you have research experience prior to dental school?** ❑ Yes ❑ No

If **yes**, was it in a dental setting? (please specify type)
❑ Yes, in dental; experience: ____

❑ No, not in dental; experience: ____

21. **Have you had a job in the past year while in dental school?** ❑ Yes ❑ No

If **yes**, was it in a dental setting? (please specify type)
❑ Yes, in dental; experience: ____

❑ No, not in dental; experience: ____

22. **Have you been involved in a research experience in the past year while in dental school?** ❑ Yes ❑ No

If **yes**, was it in a dental setting? (please specify type)
❑ Yes, in dental; experience: ____

❑ No, not in dental; experience: ____

23. **What is your current Dental School GPA?**
- ❑ 2.75 - 3.0
- ❑ 3.0 - 3.25
- ❑ 3.25 - 3.5
- ❑ 3.5 - 4.0
- ❑n/a or unknown

24. **What is your current Dental School Class Rank?**
- ❑ 0-10
- ❑ 11-20
- ❑ 21-30
- ❑ 31-40
- ❑ 41-50
- ❑ 51-60
- ❑ 61-70
- ❑ 71-80
- ❑ 81-90
- ❑n/a or unknown
