# Supplementary material for: Change is never easy: Exploring the transition from undergraduate to dental student in a U.S.-based program
Source: PLoS One. 2025 Apr 15;20(4):e0321494. doi: 10.1371/journal.pone.0321494 (PMC11999116; doi:10.1371/journal.pone.0321494)
Supplement: S3 File — (PDF) [file pone.0321494.s003.pdf]

Appendix C: Survey - Dental Students' Perception of Undergraduate and Pre-doctoral Experience Dental Student (second, third and fourth year dental students in the second year of the study and moving forward) (Class of 2019/2194-Spring 2019)

The goal of this study is to help students at dental schools have more realistic expectations and be better prepared for the academic expectations and experience during their education in the dental pre-doctoral program. Based on the results from this study, faculty and administrators at dental schools will be able to identify areas in which schools can provide support to the struggling students to adapt to the expectations in the dental school environment. Responses will be tracked longitudinally via the anonymous unique identifier. This study was approved by the University of Pittsburgh IRB (IRB #: PRO15070414) on 8/13/2015.

Anonymous Unique Identifier
First two letter of you mother's maiden (sur)name: ___ ___
Day of the month you were born (add leading zero if it's one digit): ___ ___
Example: Mary Smith (maiden name: Mary Kline): **K L**
March 4, 1995: **0 4**

---

1. **What are the most important differences you have experienced between your undergraduate classes and dental school classes?**
____
____
____

2. **What are the most important differences you have experienced between your undergraduate instructors and your dental school instructors?**
____
____
____

3. **What are the most important differences you have had between your undergraduate experiences and your dental school experiences that are not related to classes?**
____
____
____

4. **How would you rate your preparedness for dental school?**
❑ Very adequate
❑ Somewhat adequate
❑ Somewhat inadequate
❑ Very inadequate

5. **How would you rate the work load in dental school compared to undergraduate?**
❑ Much More
❑ More
❑ About the same
❑ Less
❑ Much less

6. **How would you rate your ability to manage your time in dental school compared to undergraduate?**
❑ Much better
❑ Better
❑ About the same
❑ Worse
❑ Much worse

7. **How would you rate your stress level in dental school compared to undergraduate?**
❑ Much higher
❑ Higher
❑ About the same
❑ Lower
❑ Much Lower

8. **How would you rate the academic support system in dental school compared to undergraduate?**
❑ Much better
❑ Better
❑ About the same
❑ Worse
❑ Much worse

Please turn over ➔

9. **Have you had a job in the past year while in dental school?** ❑ Yes ❑ No

If yes, has it been in a dental setting? (please specify type)
❑ Yes
❑Dental
Experience___________________________________

If yes, but *not* in a dental setting, what has been the experience? (please be specific)

❑ Other
Experience___________________________________

10. **Have you been involved in a research experience in the past year while in dental school?** ❑ Yes ❑ No

If yes, has it been in a dental setting? ❑ Yes ❑ No
(please specify type)

❑ Dental
Experience___________________________________

If yes, but *not* in a dental setting, what has been the experience? (please be specific)

❑ Non-Dental
Experience___________________________________

11. **What is your current Dental School GPA?**

❑ 2.75 - 3.0
❑ 3.0 - 3.25
❑ 3.25 - 3.5
❑ 3.5 - 4.0
❑n/a or unknown

12. **What is your current Dental School Class Rank?**

❑ 0-10
❑ 11-20
❑ 21-30
❑ 31-40
❑ 41-50
❑ 51-60
❑ 61-70
❑ 71-80
❑ 81-90
❑n/a or unknown

If you are an **advanced standing student**, please answer the following questions:

Anonymous Unique Identifier
First two letter of you mother's maiden (sur)name: ___ ___
Day of the month you were born (add leading zero if it's one digit): ___ ___
Example: Mary Smith (maiden name: Mary Kline): **K L**
March 4, 1995: **0 4**

---

13. **What was your overall undergraduate GPA (ECE)?** ❑ 2.75 - 3.0 ❑ 3.25 - 3.5 ❑ 3.0 - 3.25 ❑ 3.5 - 4.0

14. **Which college did you attend for your undergraduate degree? (please provide the official name, state and country)**

____

____

____

15. **What degree(s) do you hold (mark all that apply)? If other, please specify.** ❑ BS ❑ MA ❑MsED ❑ MD ❑ BA ❑ MBA ❑ PhD ❑BDS/MDS ❑ MS ❑ MPH ❑ JD ❑other

16. **Do you have a dental professional in the family, if so what is their profession?** ❑ Yes ❑ No

❑Dentist, ❑Dental Hygienist, ❑Dental Assistant, ❑Dental Technician, ❑Other (please specify)
____

17. **At what age did you start Pitt Dental school?** ❑ < 21 ❑ 27-30 ❑ 21-23 ❑ >30 ❑ 24-26

18. **How would you rate your preparedness for Pitt dental school?**
❑ Very adequate
❑ Somewhat adequate
❑ Somewhat inadequate
❑ Very inadequate

19. **Did you have work experience prior to Pitt dental school?**
❑ Yes ❑ No

If **yes**, was it in a dental setting? (please specify type)
❑ Yes, in dental; experience: ____

❑ No, not in dental; experience: ____

20. **Did you have research experience prior to Pitt dental school?** ❑ Yes ❑ No

If **yes**, was it in a dental setting? (please specify type)
❑ Yes, in dental; experience: ____

❑ No, not in dental; experience: ____
